# Integrated metabolome and transcriptome analysis of the NCI60 dataset

**DOI:** 10.1186/1471-2105-12-S1-S36

**Published:** 2011-02-15

**Authors:** Gang Su, Charles F Burant, Christopher W Beecher, Brian D Athey, Fan Meng

**Affiliations:** 1 Bioinformatics Program, University of Michigan, Ann Arbor, MI 48109, USA; 2 University of Michigan Medical School, University of Michigan, Ann Arbor, MI 48109, USA; 3 Michigan Center for Translational Pathology, University of Michigan, Ann Arbor MI 48109, USA; 4 National Center for Integrative Biomedical Informatics, University of Michigan, Ann Arbor, MI 48109, USA; 5 Psychiatry Department and Molecular Behavioral Neuroscience Institute, University of Michigan, Ann Arbor, MI 48109, USA

## Abstract

**Background:**

Metabolite profiles can be used for identifying molecular signatures and mechanisms underlying diseases since they reflect the outcome of complex upstream genomic, transcriptomic, proteomic and environmental events. The scarcity of publicly accessible large scale metabolome datasets related to human disease has been a major obstacle for assessing the potential of metabolites as biomarkers as well as understanding the molecular events underlying disease-related metabolic changes. The availability of metabolite and gene expression profiles for the NCI-60 cell lines offers the possibility of identifying significant metabolome and transcriptome features and discovering unique molecular processes related to different cancer types.

**Methods:**

We utilized a combination of analytical methods in the R statistical package to evaluate metabolic features associated with cancer cell lines from different tissue origins, identify metabolite-gene correlations and detect outliers cell lines based on metabolome and transcriptome data. Statistical analysis results are integrated with metabolic pathway annotations as well as COSMIC and Tumorscape databases to explore associated molecular mechanisms.

**Results:**

Our analysis reveals that although the NCI-60 metabolome dataset is quite noisy comparing with microarray-based transcriptome data, it does contain tissue origin specific signatures. We also identified biologically meaningful gene-metabolite associations. Most remarkably, several abnormal gene-metabolite relationships identified by our approach can be directly linked to known gene mutations and copy number variations in the corresponding cell lines.

**Conclusions:**

Our results suggest that integrative metabolome and transcriptome analysis is a powerful method for understanding molecular machinery underlying various pathophysiological processes. We expect the availability of large scale metabolome data in the coming years will significantly promote the discovery of novel biomarkers, which will in turn improve the understanding of molecular mechanism underlying diseases.

## Background

Metabolites are end products of cellular processes. Thus the profile of metabolites provides a snapshot of the physiological state of a cell complementary to its transcriptome and proteome, which manifests the functional status of events which occur more upstream. Since the size of metabolome is about 1-2 orders of magnitude smaller than transcriptome and proteome, the steady state concentration of a metabolite usually reflects the combined effects of multiple upstream factors. Conceivably, this property makes metabolites better biomarkers in some situations. In addition, identifying interrelations between metabolites and genes, proteins, or genome structures can potentially facilitate the elucidation of molecular mechanisms involved in pathophysiological processes.

Previous work has revealed significant associations between gene and metabolite expression profiles, which added another layer of quantitative inferences to gene-wise correlations [1-3]. Nam et. al demonstrated that the integrative study of transcriptomics and metabolomics could effectively identify metabolic biomarkers for breast cancer [4]. Nevertheless, the noisy nature of the metabolome data, limited number of metabolites with known structures in metabolome data, the lack of annotation of metabolite-gene relationships despite databases like EHMN and BiGG [5,6], the indirect nature of potential gene-metabolite relationships, and the lack of large scale metabolome study data in the public domain, still limit our knowledge of metabolites and their regulation in normal and disease processes.

Conceivably, cancer samples provide excellent opportunities for identifying metabolic biomarkers and gene-metabome relationships due to the dramatic function alterations at the molecular level in cancer tissues. For example, cancer tissues usually exhibit more than 10-fold changes in the expression level of many genes in numerous microarray studies. Recently, the collaborative NCI60 project from the Developmental Therapeutics Program (DTP) of the National Cancer Institute (NCI) has made extensive measurements of various ‘omics’ data publicly available, including microarray, metabolomics, proteomics, etc. While the number of metabolome of metabolome-related studies and biomarker discoveries associated with cancer is increasing rapidly in targeted studies, there is still no literature on comprehensive analysis of metabolic features and their regulatory mechanisms of different cancer types across multiple ‘omics’ data.

In this study, we want to investigate whether different cancer cell lines have distinct metabolic signatures and whether available metabolome data for the NCI-60 cell lines is suitable for cancer subtype classification. We also hope the combined metabolome and transcriptome analysis will reveal some distinct regulatory relationships in some of the NCI-60 dataset which are not otherwise possible by either metabolome or transcriptome study alone. We expect the analysis approach used in this work can be applied to other metabolome-transcriptome datasets when they become available.

## Results

### Metabolomic signature of cancer cells from different tissue origins

The first question we would like to address is whether cancer cell lines exhibit tissue-origin specific metabolic signatures. We used classification analysis similar to those performed in microarray data [7] to classify 57 cell-lines into 9 cancer types related to their tissue origins. Our initial tests suggest that regardless of method used, the classification error on metabolomics data is always significantly higher than that from microarray (~0.51 for metabolomics and ~0.34 for microarray).

Besides the fact that NCI-60 metabolome dataset has much less data points but with higher noise levels when compared to microarray data, we speculate that the low number of some cancer types (e.g. only 2 prostate cancer cell lines) and high heterogeneity of some cell lines (breast, ovarian and CNS) may also undermine the performance of the metabolome-based classification. In fact, these 4 cell lines contribute most to the out of bag classification error estimates.

**Figure 1:**
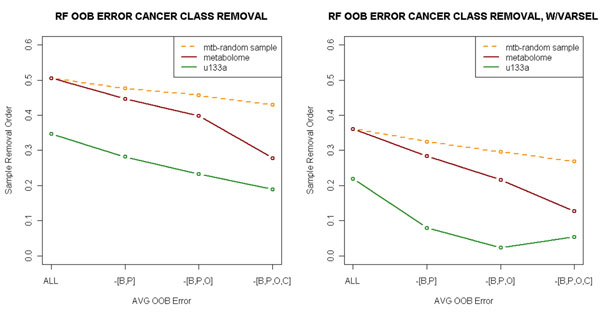
**Classification error.** Estimated Out-of-Bag (OOB) Error with regard to progressive cancer class removal. At each step, cancer classes contribute most to classification error were removed and OOB errors were recalculated. B: Breast Cancer. P: Prostate Cancer. O: Ovarian Cancer. C: CNS. The left plot shows OOB errors on entire dataset and the right plot shows OOB errors on subset of classifiers selected by varSelRF. The improvement of variable selection is more remarkable for microarray because of the much larger classifier pool to select from (11961 v.s. 342). Metabolite classifiers can achieve average OOB error of 0.28 when B,P,O,C are removed, reduced from 0.51 from the full set. The OOB error for u133a reduced from 0.34 to 0.18. The average OOB errors from the random cancer class removal are plotted in the orange dashed line.

After prostate, breast, ovarian and CNS cell lines are removed from the whole sample, the metabolite classifiers can reach comparable performance with microarray classifiers (Figure 1). The significantly improved metabolome-based classification results after removing cell lines with high variability or heterogeneity strongly suggests that there are indeed cancer subtype-specific (based on tissue of origin) metabolic signatures.

Since the removal of cancer classes will also reduce the complexity of the data thus naturally improve classification performance, we also removed the same number of cancer classes at each stage randomly for 500 times to estimate the effect of improvement of OOB errors due to simplification of data structure resulted from sample removal (orange dashed lines in Figure 1). It can be seen that the reductions in OOB errors from random removal are not as significant as those from progressively removal of the prostate, breast, ovarian and CNS cell line classes, suggesting that it is indeed the high heterogeneity and the low sample number of these cell line classes that caused the high OOB error in the original classification. Consequently, our results suggest that although metabolite profiles are not as good as microarray data for the classification of cancer cell lines, metabolites nonetheless contain cell type specific signature and the classification results can be improved if we have more samples or have more data points in the metabolome assays.

### Correlation analysis

Although existing metabolome data do not outperform microarray data in cell line subtype classification, metabolome can still be useful for revealing some basic molecular processes and their alterations in NCI-60 cell lines. We hypothesize that different cell lines should share some basic regulatory and metabolic processes (steady-state) essential for cell growth and metabolism. It is likely that although different cancer cell lines may have very different levels of gene expression and metabolism, the steady-state relationship between genes and metabolites in the same or highly coupled pathways should be conserved across different cell lines in the absence of dramatic genomic changes such as gene mutation and copy number variation (CNV). Identification of such gene-metabolite relationships at steady state will help us better understand the underlying molecular mechanisms related to the metabolic change. They will also help us infer potential metabolic changes based on the expression data or vice versa. On the other hand, if a few cell lines deviated significantly from the gene-metabolite relationships exhibited by the most of the cell lines, the related genes in such outlier cell lines are likely to be silenced (no expression) or agitated (over expression) due to genomic structural changes. Consequently, we are interested in both high correlations which reflect steady state trend over all samples, and outliers which reflect signatures in specific cell lines.

The classic method of inspecting the relatedness of two quantitative features is by computing the Pearson Correlation Coefficient (PCC). While PCC have been proven to work well in many gene expression studies, the correlation is often inflated by outliers or clustering effect, which made it not suitable to analyze data with high noise level such as metabolic profiles. In contrast, the robust correlations can provide a much better estimate of the true association in the presence of multi-dimensional outliers if the sample size is sufficiently large. We chose the Pair-wise Quadrant Correlation (PQC) for its good computational performance. However, for small sample size, PQC sometimes may inflate the correlation estimate. Therefore our correlation analysis, we use a novel approach by combining results from both PCC and PQC. Gene-metabolite pairs with high PCC and high PQC tend to demonstrate true linear correlation across all cancer types, which imply a general functional association between gene and metabolite profiles. On the other hand, gene-metabolite pairs with high PCC and low PQC are possibly resulted from a few extreme outliers, which can potentially be linked to cell-line specific signatures.

### Metabolite-gene correlations

Since metabolite-gene relationships can help us to understand the direct or indirect cause of metabolite changes, we computed PCC and PQC for 11872 gene expression profiles and 253 metabolite measurements over 57 cell-lines.

To investigate the biological significances of the correlation analysis, we mapped all the known gene-compound associations from EHMN to our NCI60 analysis. There are a total of 721 EHMN annotated direct metabolite-gene associations, involving 352 genes and 81 known metabolites, that overlap with our NCI-60 metabolite-gene relationships.

**Figure 2:**
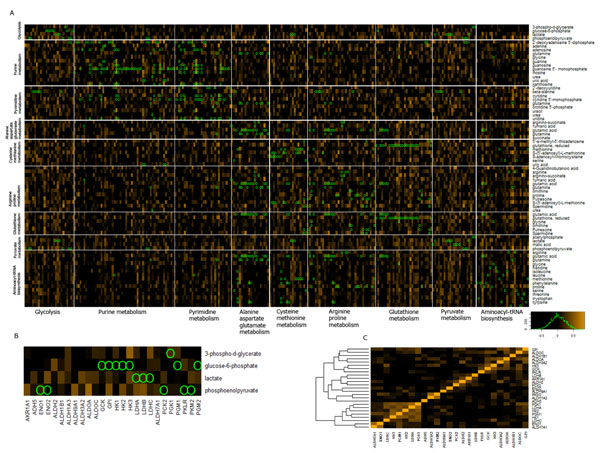
**Heatmap of gene-metabolite relationship organized according to KEGG pathway**. Mapping of EHMN gene-metabolite association data to robust correlation matrix heat map. A. Rows are genes grouped by pathway names, columns are metabolites also grouped by corresponding pathway names. Green circles mark the position of a specific reaction that couples a metabolite and a gene from EHMN. Orange Light cells indicate positive PQC, with black to be 0 and orange to be 1. It can be seen that even though there are patterns of gene-metabolite clustering, very few high correlations can be mapped to known reactions. B. Gene-metabolite correlation in the Glycolysis pathway. C: The gene-gene correlation with in Glycolysis pathway.

Figure 2 shows a mapping of our current metabolite-compound knowledgebase to the robust correlations. We chose the top 9 pathways in EHMN mapping ordered by number of genes

Some of the EHMN gene-compound relationships do show high association across all cell-lines. For example, gene AKR1B1 which reduces L-Arabitol to L-Arabinose with EHMN reaction ID R01758 and R01759, is associated with L-Arabitol with PQC of 0.69 and PCC of 0.36, which implies strong association between metabolite level and gene activity. However, it is surprising that most of the direct enzyme-metabolite relationships cannot be mapped to high correlations (Figure 2A and 2B). There are several possible explanations to the low match-ups of significant correlations to EHMN data. A simple explanation is most of the annotatable gene-metabolite relationships in the NCI-60 dataset may not be the speed limiting factor in the related pathways. It is also possible that the high level of abnormal regulation in the cancer cell lines may have masked global mechanisms such that it is difficult to identify high gene to metabolite correlations across all cell lines.

Interestingly, the grouping of genes in the same pathway in Figure 2 enables us to detect many metabolites exhibit high correlations with other genes in the same pathway. For example, although phosphoenolpyruvate does not overlap with any of the EHMN annotated direct reaction genes in Figure 2B, it has high correlation with GPI and ALDOA, two of the genes in the glycolysis module that are known to be highly regulated by hypoxia-inducible factor 1alpha and such regulation is related to the aggressive phenotype of hepatocellular carcinoma. Consequently, ALDOA, GPI and other genes highly correlated with phosphoenolpyruvate in our multi-cancer cell line analysis may suggest that these genes has more significant regulatory or speed limiting roles in glycolysis than genes such as ENO1, ENO2 that are directly related to reactions involving phosphoenolpyruvate in these cancer cell lines. Naturally, not all genes in the same pathway strongly correlate with each other since genes in the same pathway are not all (Figure 2C).

Further investigation will be worthwhile for high correlation between metabolites and other genes (i.e. those not directly involved in the specific metabolic reaction) in the same pathway, as such un-annotated relationships are likely help us to identify speed limiting enzymes in a pathway, key regulatory genes of related pathways or novel metabolic mechanisms.

### Outlier analysis

In our previous analysis we require high PQC in addition to high PCC to identify molecule pairs with true high correlation. We have also identified many cases where cell lines have one or a few gene-metabolite pairs with much higher expression values than the rest of the other samples, which directly produce inflated PCC and very low PQC. To systematically investigate these cases, we used R package mvoulier to detect multidimensional outliers, and recomputed the PCC and PQC scores after outlier removal. Our empirical rule shows that when PCC > 0.6 and PQC < 0.3(Preferably close to 0), and the number of multidimensional outliers is smaller than 3, the high PCC is most likely to be an artifact from very few extreme outliers.

**Figure 3:**
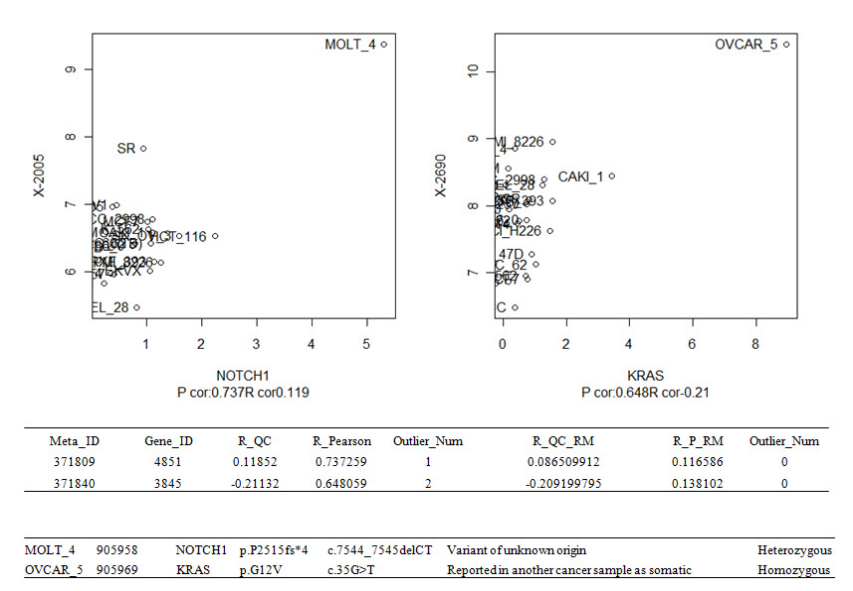
**Outlier analysis.** Outlier analysis shows extreme gene-metabolite pairs could be resulted from cell specific mutations. Top scatter plots: NOTCH1 ~ X-2005, with outlier in cell line MOLT_4 and KRAS ~ X-2690, with outlier in OVCAR5. It can be seen that the high PCC were both artefacts of extreme outliers. Middle table: PCC and PQC of the corresponding pairs, before and after outlier removal. R_QC: PQC. R_QC_RM: PQC after outlier removal. P_Pearson: PCC. R_P_RM: PCC after outlier removal. Bottom table: annotated mutation records from Sanger Cosmic database, directly matched to these two outlier pairs.

To explore the biological significance of these outliers, we compared the outlier cases detected by the criteria mentioned above to the Sanger Cosmic database [8]. Sanger Cosmic contains 177 mutation entries of 28 genes in our 57 NCI60 cell-lines. We ordered all the metabolite-gene correlations of the 28 genes by PCC, and amazingly the top two outliers detected by our approach, NOTCH1~X-2005 in MOLT-4 cell line and KRAS~in X-2690 in OVCAR-5 cell line, are the cell lines with known gene mutation on the exact same genes in Sanger Cosmic database (Figure 3).

The common feature of these two gene-metabolite pairs is high PCC and low PQC before outlier removal and low PCC and low PQC after the outlier removal. From plots in Figure 3 we can verify that the inflated PCC were indeed a product of single cell line outlier. Besides, the sample sizes of these two cases are sufficiently large (22 for NOTCH1~X-2005 and 26 for KRAS~X-2690, respectively) so that the outlier is not likely to be a random fluctuation from small sample size.

Since our unbiased analysis did not take advantage of any cell line or gene mutation information, the fact that our top two outliers overlaps with documented gene mutation in the Sanger Cosmic dataset suggests that the mutations are likely to be a cause of such outliers, and the associated metabolites may be good candidate biomarkers for such events.

In addition to point mutations, we also compared the outlier analysis with Copy Number Variation (CNV) data from Broad institute. Only 34 out of 57 samples from NCI60 have CNV data, and also found some gene-metabolite outlier pairs that are consistent with CNV outliers. For example, gene BRIP1, with CNV of 14.22 in cell line MCF7, has PCC of 0.90 and PQC of 0.008 and one outlier. The corresponding compound, X-3363, may be associated specifically to this copy number variation.

The fact that some top ranked gene-metabolite outlier associations matches with known corresponding genomic structural changes in the specific gene strongly suggests the effectiveness of our approach in identifying distinct molecular processes specific to metabolome and transcriptome variations.

## Methods

NCI-60 data pre-processing: raw molecular datasets were downloaded from DTP web portal (http://dtp.nci.nih.gov/index.html March 2007 release). There are 57 cell-lines in 9 cancer classes have both microarray and metabolomics data. We used our in-house Entrez-based Custom CDF version 12 to derive gene-level expression data from the NCI-60 Affymetrix Genechip CEL files (http://brainarray.mbni.med.umich.edu)[9]. The metabolite data averaged over triplicate experiments were manually compared with a reference dataset to exclude imputed values (Beecher, unpublished data) as the imputed data could significantly bias the inferences drawn from correlation analysis. All metabolite names were manually compared with KEGG and assigned a KEGG compound ID whenever possible. The pre-processing step produced 11961 and 6089 gene expression profiles from Affymetrix HG-U133A and HG-U133B chips respectively, and quantitative data for 124 known and 218 unknown metabolites.

All statistical analyses were performed in R (http://www.r-project.org/). Classification and variable selection were performed by R randomForest (http://cran.r-project.org/web/packages/randomForest/index.html) and varSelRF package [10]. The robust correlations were computed with robust package (http://cran.r-project.org/web/packages/robust/index.html), using the parameter pair-wise Quadrant Correlation (pairwiseQC). Multidimensional outliers were identified by package mvoutlier [11]. We performed the computation on our LINUX cluster of ~100 cores. The results were loaded into an Oracle database for integrative analysis.

The annotated gene to metabolite relationship data kindly offered by Edinburgh Human Metabolic Network project is used for identifying biological relevant known gene-metabolite relationships revealed by our analysis [5]. We also compiled a local version of KEGG [12] and DAVID 2008 [13] for similar purposes. In order to associate known mutations and CNVs in NCI-60 cell lines to abnormal gene-metabolite relationships, we have built a local copy of COSMIC [8] dataset from Sanger and Tumorscape [14] from Broad institute for cell-line specific point-mutation and copy number variations (CNV), respectively.

## Discussion

Our analysis results on the NCI-60 metabolome and transcriptome data suggest that 1) although the small sample size, the high noise level and intra-cancer class heterogeneity in the current NCI-60 metabolome dataset makes it unsuitable for global cancer subtype classification, there are indeed metabolic signatures associated with cancer subtypes. 2) There are biologically meaningful high correlation gene-metabolite pairs across NCI-60 cell lines, identifiable by robust correlation estimates. 3) Most strikingly, there are several examples of abnormal gene-metabolite coupling that can be directly linked to known gene mutations or copy number variations.

Conceivably, high correlations as well as outliers can be utilized to aid the progressive prediction of unknown metabolites based on annotations in existing pathway databases and literature. For example, the high correlation of an unknown compound with a known gene and in particular, multiple known genes in a pathway can dramatically reduce the search space for the unknown compound, since the most likely candidates will be structurally related molecules or known metabolites from related pathways. We plan to compare the Mass Spectrometer (MS) features of these unknown compounds from the predicted candidate pool and conduct wet-lab experiments for validation. Since we have discovered that many unknown metabolites are strongly associated with each other but not with any known compounds. Correct determination of even a small fraction of them would facilitate the identification of the rest, which also will in turn improve our understanding of the molecular processes and pathways involving these molecules.

In summary, the strong biological relevance of our results also suggest that the analysis strategy we developed based on the combination of PCC and PQC correlation values for identifying real correlation and true outliers is a powerful approach for integrative analysis of noisy ‘omics’ datasets. The presentation of gene-metabolite relationship analysis results in a heatmap grouped by genes in the same pathway together with overlay of known gene-metabolite relationship provides a powerful visual exploration approach for identifying both direct and indirect gene-metabolite relationships. The analysis methods we described here will be useful for other integrated analysis of metabolome and transcriptome and the wet lab validation of novel gene-metabolite relationships.

## Competing interests

The authors declare that they have no competing interests.

## Authors' contributions

GS performed data cleaning, correlation analysis and outlier analysis. CFB helped with correlation analysis and functional inference. CWB cleaned metabolomics data and annotated missing values. GS, BDA evaluated robust correlation methods; FM initialized the study, compiled microarray data and supervised biological validation.

## References

[B1] Ferrara CT Wang P Neto EC Stevens RD Bain JR Wenner BR Ilkayeva OR Keller MP Blasiole DA Kendziorski C Genetic networks of liver metabolism revealed by integration of metabolic and transcriptional profiling PLoS Genet 2008 4 e1000034 10.1371/journal.pgen.1000034 18369453 PMC2265422

[B2] Xu EY Perlina A Vu H Troth SP Brennan RJ Aslamkhan AG Xu Q Integrated pathway analysis of rat urine metabolic profiles and kidney transcriptomic profiles to elucidate the systems toxicology of model nephrotoxicants Chem Res Toxicol 2008 21 1548 1561 10.1021/tx800061w 18656965

[B3] Carrari F Baxter C Usadel B Urbanczyk-Wochniak E Zanor MI Nunes-Nesi A Nikiforova V Centero D Ratzka A Pauly M Integrated analysis of metabolite and transcript levels reveals the metabolic shifts that underlie tomato fruit development and highlight regulatory aspects of metabolic network behavior Plant Physiol 2006 142 1380 1396 10.1104/pp.106.088534 17071647 PMC1676044

[B4] Nam H Chung BC Kim Y Lee K Lee D Combining tissue transcriptomics and urine metabolomics for breast cancer biomarker identification Bioinformatics 2009 25 3151 3157 10.1093/bioinformatics/btp558 19783829

[B5] Ma H Sorokin A Mazein A Selkov A Selkov E Demin O Goryanin I The Edinburgh human metabolic network reconstruction and its functional analysis Mol Syst Biol 2007 3 135 10.1038/msb4100177 17882155 PMC2013923

[B6] Schellenberger J Park JO Conrad TM Palsson BO BiGG: a Biochemical Genetic and Genomic knowledgebase of large scale metabolic reconstructions BMC Bioinformatics 2010 11 213 10.1186/1471-2105-11-213 20426874 PMC2874806

[B7] Wang H Huang S Shou J Su EW Onyia JE Liao B Li S Comparative analysis and integrative classification of NCI60 cell lines and primary tumors using gene expression profiling data BMC Genomics 2006 7 166 10.1186/1471-2164-7-166 16817967 PMC1525183

[B8] Forbes SA Tang G Bindal N Bamford S Dawson E Cole C Kok CY Jia M Ewing R Menzies A COSMIC (the Catalogue of Somatic Mutations in Cancer): a resource to investigate acquired mutations in human cancer Nucleic Acids Res 2010 38 D652 657 10.1093/nar/gkp995 19906727 PMC2808858

[B9] Dai MH Wang PL Boyd AD Kostov G Athey B Jones EG Bunney WE Myers RM Speed TP Akil H Evolving gene/transcript definitions significantly alter the interpretation of GeneChip data Nucleic Acids Res 2005 33 e175 10.1093/nar/gni179 16284200 PMC1283542

[B10] Diaz-Uriarte R GeneSrF and varSelRF: a web-based tool and R package for gene selection and classification using random forest BMC Bioinformatics 2007 8 328 10.1186/1471-2105-8-328 17767709 PMC2034606

[B11] Filzmoser P Maronna R Werner M Outlier identification in high dimensions Comput Stat Data An 2008 52 1694 1711 10.1016/j.csda.2007.05.018

[B12] Kanehisa M The KEGG database Novartis Found Symp 2002 247 91 101 discussion 101-103, 119-128, 244-152full_text 12539951

[B13] Dennis G Jr. Sherman BT Hosack DA Yang J Gao W Lane HC Lempicki RA DAVID: Database for Annotation, Visualization, and Integrated Discovery Genome Biol 2003 4 P3 10.1186/gb-2003-4-5-p3 12734009 PMC193660

[B14] Beroukhim R Mermel CH Porter D Wei G Raychaudhuri S Donovan J Barretina J Boehm JS Dobson J Urashima M The landscape of somatic copy-number alteration across human cancers Nature 2010 463 899 905 10.1038/nature08822 20164920 PMC2826709

